# Effect of Different Factors on the Short-Term Outcome of Chinese Patients With Primary Chronic Subdural Hematoma at Different Age Groups: A Two-Center Retrospective Study

**DOI:** 10.3389/fnagi.2019.00325

**Published:** 2019-11-29

**Authors:** Jinhao Huang, Ye Tian, Yiming Song, Rong Hu, Shuixian Zhang, Zhitao Gong, Xuanhui Liu, Hongliang Luo, Chuang Gao, Dong Wang, Hua Feng, Jianning Zhang, Rongcai Jiang

**Affiliations:** ^1^Department of Neurosurgery, Tianjin Medical University General Hospital, Tianjin, China; ^2^The State Key Laboratory of Neurotrauma Repair and Regeneration, Ministry of Education and Tianjin City, Tianjin, China; ^3^Key Laboratory of Neurotrauma, Department of Neurosurgery, Southwest Hospital, Third Military Medical University (Army Medical University), Chongqing, China

**Keywords:** epidemiology, outcome features, chronic subdural hematoma, different ages, atorvastatin

## Abstract

**Background:**

The incidence of symptomatic chronic subdural hematoma (CSDH) is increasing in Chinese aging population, but its clinical and demographic knowledge is still lacking. This study sought to outline the clinical and demographic data of CSDH patients from two medical centers in Tianjin and Chongqing to provide a better understanding for CSDH treatment in China.

**Methods:**

Age, sex, etiologies, conscious level on admission, treatment strategies, outcome at discharge, recurrence, and concomitant disease of enrolled patients were recorded. The data were further analyzed after the patients were sub-grouped into young/middle (less than 60 years old) and old (over than 60 years old) age groups.

**Results:**

A total of 1281 CSDH patients were enrolled. Of these, 85.01% were male and 51.91% were aged between 60 and 80 years. 57.61% patients encountered head trauma before diagnosing CSDH. The top three clinical symptoms at admission were headache (58.55%), dyskinesia (36.92%), and dizziness (33.96%). Headache as well as dizziness often happened in young/middle age group, while dyskinesia often occurred in the old age group. The most common concomitant diseases were cardio-cerebrovascular system diseases (41.14%). The concomitant respiratory diseases in aged patients led to unfavorable outcomes (*p* = 0.049, OR:0.357). The prognosis of old age subgroup receiving conservative treatment was better than those who received burr-hole drainage treatment (*p* < 0.015, OR:4.091).

**Conclusion:**

CSDH mostly occurs in aged and male population with a history a head trauma. The respiratory disease often results in unfavorable outcomes in aged patients. Conservative treatment might benefit some patients.

## Introduction

Chronic subdural hematoma (CSDH) is a common disorder in neurosurgical practice, and is mostly seen in elderly patients. Its overall incidence is about 5/100,000/year in general population (Santarius and Hutchinson, 2004) and at least 8.2/100,000/year in the population over 70 years of age (Adhiyaman et al., 2002). With the increasing aged population, the population over 65 years of age is expected to double during the next few years in the world, and with steady increase of the incidence of CSDH (Lind et al., 2003). To the best of our knowledge, there were very few demographic descriptions about Chinese CSDH patients either in Chinese or English text book of neurosurgery. In addition, according to Youmans and Winn Neurological Surgery, although the burr-hole drainage is recognized as the first-line treatment for CSDH in most of countries, its recurrence rate still varied from 20 to 26% in different reports (Berghauser Pont et al., 2012; Almenawer et al., 2014; Liu et al., 2014; Ivamoto et al., 2016) independent of the surgical methods. On the other hand, several conservative treatments including the atorvastatin therapy has been developed in recent years (Holl et al., 2018), which is a new therapeutic strategy for CSDH. But the impact of factors on the therapeutic outcomes of CSDH is also still unknown. However, most of the published literatures focus either on the therapies of CSDH (Lu et al., 2018; Srivastava et al., 2018; Xu et al., 2018; Yuan et al., 2018) or on rare case reports (Mikkelsen et al., 2017; Tempaku, 2017; Ichinose et al., 2018; Wang and Yu, 2018), especially in Chinese studies. Hence, a high quality retrospective study on Chinese CSDH patients is urgently needed.

Therefore, we collected the consecutive data of CSDH inpatients admitted during January 2004 to December 2016 in both Tianjin Medical University General Hospital and Third Military Medical University Southwest Hospital. These two hospitals were localized in North China and Southwest China, respectively, representing the most advanced and the largest medical center in their areas. This study aimed at outlining an updated information on the clinical and demographic features of CSDH in Tianjin and Chongqing to help improve a better understanding for CSDH treatment in China.

## Materials and Methods

### Participants and Study Settings

**FIGURE 1:**
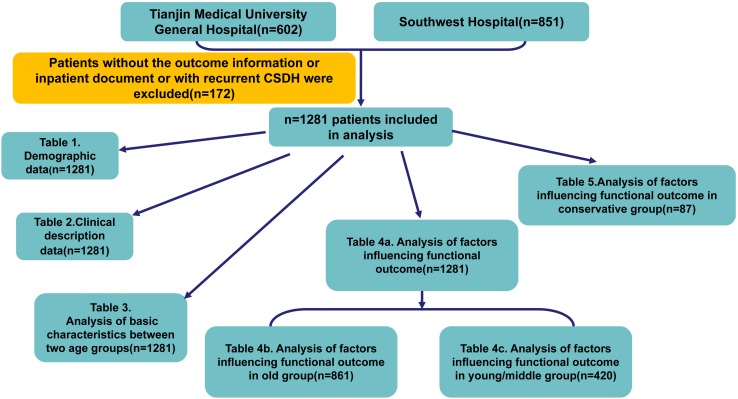
Flowchart of detailed design for the study.

This study has been approved by the Ethical Committees in both hospitals. All CSDH medical records belonged to designed period were found for this study. The recruiting criteria for factors analysis were as follows: patients (1) presenting primary CSDH as diagnosed by neuroimaging [Computed Tomography (CT) or Magnetic Resonance Imaging (MRI)], (2) aged greater than 18 years; (3) more than 2 weeks hospitalization. Patients with recurrent CSDH or without complete inpatient records and outcome information were excluded. The recurrence was defined as symptomatic and radiologically confirmed recurrent CSDH within 6 months. The patients were then sub-grouped as young/middle age group (i.e., <60 years) and old age group (≥60 years). The detailed analysis design was showed by Figure 1.

### Data Collection

A protocol for clinical data collection was followed by the two teams in both hospitals. House staff participated in medical charter review and data collection. After validation by an independent clinical research coordinator, the data collected were analyzed by the principal investigators and their teams in collaboration with biostatisticians who were blind to the study. Then the demographic data and the clinical features of these inpatients were recorded, including the medical history, gender, age, etiology, clinical symptoms, level of conciseness, location of hematoma and concomitant diseases at admission, therapeutic recording and outcomes on the day of discharge.

### Neurological Outcome Evaluation

Both the Glasgow coma score (GCS) and the modified Rankin Scale (mRS) during admission or discharge were used to evaluate the level of conciseness and the neurological function. The favorable neurological outcome was defined as mRS score 0–2 and the unfavorable score as mRS 3–6.

### Data Analysis

The quantitative data were presented as mean ± SD and the enumeration data were recorded as the number. The descriptive analysis was used on the demographic data. The Student’s unpaired *t* test or Chi Square test (χ2 test) were used to compare the variables between the groups. Univariable and multivariable logistic regression models were built to evaluate the impact of variable factors on the neurological outcome. Because of the mutual influence of various factors, our discussion was mainly based on the results of multivariable analysis. Data were analyzed by IBM SPSS 22.0 and *p* values < 0.05 was considered to be statistically significant.

## Results

### General Demographic Data

A total of 1453 patients with CSDH were recruited from two hospitals: 602 from Tianjin Medical University General Hospital and 851 from Third Military Medical University Southwest Hospital, respectively. Among these patients, 172 patients with lack of outcome information or inpatient document or with recurrent CSDH were excluded. Finally, 1281 patients entered into statistical analysis. The mean age of these patients was 64.60 years (range: 18–95 years), with 85.01% males. The percentage of patients who were between 60 and 80 years was 51.91%. The highest male ratio was as high as 91.47% in patients of 40–49 years old, but the lowest was only 68.42% in patients >90 years. Patient characteristics were detailed in Table 1.

**TABLE 1:** Demographic characteristics of 1281 patients.

**Age group**	**Male, *n* (%)**	**Female, *n* (%)**	**Male-female ratio**	**Average age (mean ± SD)**	**Percentage of total, *n* (%)**
>90	13(68.42%)	6(31.58%)	2.17	91.63 ± 1.49	19(1.48%)
80–89	143(80.79%)	34(19.21%)	4.21	82.89 ± 2.77	177(13.82%)
70–79	273(81.49%)	62(18.51%)	4.40	74.33 ± 2.78	335(26.15%)
60–69	280(84.85%)	50(15.15%)	5.60	64.55 ± 2.81	330(25.76%)
50–59	202(90.99%)	20(9.01%)	10.10	55.00 ± 2.84	222(17.33%)
40–49	118(91.47%)	11(8.53%)	10.73	44.98 ± 2.81	129(10.07%)
30–39	35(87.50%)	5(12.50%)	7.00	35.53 ± 2.58	40(3.12%)
18–29	25(86.21%)	4(13.79%)	6.25	24.21 ± 3.25	29(2.26%)
In total	1089(85.01%)	192(14.99%)	5.67	64.60 ± 14.41	1281(100.00%)

*All results are given as number (%) or mean ± SD. *n*: number of cases; SD: standard deviation.*

### Clinical Symptoms, Etiology and Features of Hematomas

Headache (58.55%) was the most frequent clinical symptom observed in all patients during admission, followed by dyskinesia (36.92%) and dizziness (33.96%). The head neuroimaging showed that 1001 (78.14%) patients had suffered from unilateral hematomas and only 280 (21.86%) had bilateral hematomas. Except less than 1% of patients who had poor conscience with GCS of 3∼8, the remaining patients were admitted with a GCS of more than nine at admission. However, when assayed with mRS, 58.55% of patients suffered from significant neurological defects, and had a score of mRS 3∼5 at admission (Table 2).

**TABLE 2:** Variable characteristics of 1281 patients.

**Characteristics**	**No. of patients (*n*%)**
**Main clinical symptoms at admission**	
Headache	750(58.55%)
Dyskinesia	473(36.92%)
Dizziness	435(33.96%)
Vomiting	52(4.06%)
Nausea	43(3.36%)
Hypomnesis	19(1.48%)
Epilepsy	12(0.94%)
Visual disturbance	11(0.86%)
**Etiology**	
History of head trauma	738(57.61%)
Unknown	543(42.39%)
**Distribution of hematoma**	
Unilateral hematoma	1001(78.14%)
Bilateral Hematoma	280(21.86%)
**Main concomitant diseases at admission**	
Cardio-cerebrovascular system disease	527(41.14%)
Respiratory system disease	351(27.40%)
Endocrine system disease	105(8.20%)
Malignant tumor	33(2.58%)
Hematological system diseases	11(0.86%)
Immune system disease	8(0.62%)
**Treatment**	
Burr- hole craniostomy	1132(88.37%)
Conservative treatment	87(6.79%)
Craniotomy	62(4.84%)
**Anti-thrombotic drug**	
Anti-coagulant	10(0.78%)
Anti-platelet	23(1.80%)
**GCS at admission**	
3–8	9(0.70%)
9–11	311(24.28%)
12–15	961(75.02%)
**mRS at admission**	
0–2	531(41.45%)
3–5	750(58.55%)
**Outcome**	
Favorable (mRS = 0–2)	1006(78.53%)
Unfavorable (mRS = 3–6)	275(21.47%)

*All results are given as number (%). *n*: Number of cases.*

Seven hundred and thirty-eight patients (57.61%) documented a history of head trauma in the recent 3 months, while the etiology of 543 (42.39%) patients remained unknown (Table 2). Both headache and dizziness were more frequently observed in young/middle (18–59 years old) group when compared to the old ones (older than 59 years) (80.00% vs. 48.08%, *p* < 0.001; 38.33% vs. 31.82%, *p* < 0.001, respectively). However, dyskinesia was less frequent in young/middle group than the older ones (15.95% vs. 47.15%, *p* = 0.021). The incidence of head trauma was decreased with increasing age (young/middle vs. old group: 67.86% vs. 52.61%, *p* < 0.001). The anti-platelet treatment was more prevalent in the old group compared with the young/middle group (*p* = 0.044) (Table 3).

**TABLE 3:** Basic clinical characteristics analysis in different age groups.

**Variable**		**Young/Middle group (18–59), *n* = 420**	**Old group (over than 59), *n* = 861**	***p*-value**
Main clinical symptoms at admission (*n*%)	Headache	336(80.00%)	414(48.08%)	< 0.001
	Dyscinesia	67(15.95%)	406(47.15%)	< 0.001
	Dizziness	161(38.33%)	274(31.82%)	0.021
	Vomiting	29(6.90%)	23(2.67%)	< 0.001
	Nausea	20(4.76%)	23(2.67%)	0.051
	Hypomnesis	5(1.19%)	14(1.63%)	0.545
	Visual disturbance	9(2.14%)	2(0.23%)	0.001^∗^
	Epilepsy	2(0.48%)	10(1.16%)	0.356^∗^
Anti-thrombotic drug	Anti-coagulant	6(1.43%)	4(0.46%)	0.089^∗^
	Anti-platelet	3(0.71%)	20(2.32%)	0.044^∗^
Distribution of hematoma	Bilateral	83(19.76%)	197(22.88%)	0.205
Etiology	History of head trauma	285(67.86%)	453(52.61%)	< 0.001

*All results are given as number (%). ^∗^Fisher exact test. *n*: number of cases.*

### Concomitant Diseases

The most common concomitant disease was cardio-cerebrovascular system disease (41.14%), followed by respiratory system disease (27.40%), and endocrine system disease (8.20%). The cardio-cerebrovascular system diseases included hypertension, myocardial or cerebral infarction, arrhythmia, and valvulopathy, and of which hypertension accounted for majority. The respiratory system diseases included chronic obstructive pulmonary disease (COPD), lung abscess, tuberculosis, bronchitis, and pneumonia. Most of the respiratory diseases were COPD. Almost all the patients with endocrine system diseases had diabetes, while some other patients had hyperthyroidism and gout (Table 2).

### Treatment, Outcome Characteristics and Factors Impact on the Outcome

The Burr-hole drainage was the most frequent surgical treatment for CSDH (88.37%) with 4.84% of craniotomy. The conservative treatment accounted for 6.79%. According to mRS, the favorable neurological outcome was observed in 1006 (78.53%) patients and unfavorable in 275 (21.47%).

The 1281 were then sub-grouped into favorable and unfavorable groups based on their mRS scores at discharge. The logistic regression was performed (Tables 4a–c, 5) to conclude the association among the neurological outcomes, clinical data and treatment.

**TABLE 4a:** Factors influencing functional outcomes.

**Variable (*n* = 1281)**		**Favorable mRS (0–2), (*n* = 1006)**	**Unfavorable mRS (3–6), (*n* = 275)**	**OR^∗^**	***p*-value^∗^**	**95% CI^∗^**	**OR^∗∗^**	***p*-value^∗∗^**	**95% CI^∗∗^**
Mean age years ± SD, interquartile range (years)		62.97 ± 14.09,54–74	70.58 ± 13.99,64.5–80	0.958	<0.001	0.948–0.969	0.963	0.003	0.939–0.987
Gender (male%)		870 (86.48%)	219 (79.64%)	1.636	0.005	1.159–2.309	1.302	0.528	0.574–2.95
Bilateral Hematoma		209 (20.78%)	71 (25.82%)	0.753	0.074	0.553–1.027	0.814	0.589	0.387–1.714
Average mRS at admission (*n* ± SD), interquartile range		2.43 ± 1.05,2–3	3.98 ± 0.55,4–4	0.101	<0.001	0.074–0.138	0.138	<0.001	0.079–0.243
Average GCS at admission (*n* ± SD), interquartile range		13.36 ± 1.30,12–14	9.95 ± 1.09,10–11	8.891	<0.001	6.657–11.874	7.481	<0.001	5.359–10.442
Main clinical	Headache	637 (63.32%)	113 (41.09%)	2.475	<0.001	1.885–3.249	1.221	0.566	0.617–2.418
symptoms at	Dyskinesia	336 (33.4%)	137 (49.82%)	0.505	<0.001	0.386–0.662	0.923	0.832	0.439–1.938
admission	Dizziness	363 (36.08%)	72 (26.18%)	1.592	0.002	1.181–2.144	0.836	0.618	0.413–1.691
Treatment^#^	Burr-hole craniotomy	909 (90.36%)	223 (81.09%)		<0.001	–		0.037	–
	Craniotomy	42 (4.17%)	20 (7.27%)	0.515	0.019	0.297–0.895	0.617	0.426	0.188–2.028
	Conservative treatment	55 (5.47%)	32 (11.64%)	0.422	<0.001	0.266–0.668	2.837	0.019	1.184–6.798
Etiology	History of head trauma	605 (60.14%)	133 (48.36%)	1.611	<0.001	1.232–2.106	0.999	0.999	0.537–1.861
Main	Cardio-cerebrovascular system disease	369 (36.68%)	158 (57.45%)	0.429	<0.001	0.327–0.563	1.389	0.396	0.65–2.969
concomitant	Respiratory system disease	226 (22.47%)	125 (45.45%)	0.348	<0.001	0.263–0.46	0.419	0.035	0.186–0.941
diseases at	Endocrine system disease	73 (7.26%)	32 (11.64%)	0.594	0.02	0.383–0.921	1.56	0.37	0.59–4.123
admission									

**TABLE 4b:** Factors influencing functional outcomes in old groups (*n* = 861).

**Variable (*n* = 861)**		**Favorable mRS (0–2), (*n* = 640)**	**Unfavorable mRS (3–6), (*n* = 221)**	**OR^∗^**	***p*-value*^∗^***	**95% CI^∗^**	**OR^∗∗^**	***p*-value^∗∗^**	**95% CI^∗∗^**
Mean age years ± SD, interquartile range (years)		71.55 ± 7.72,65–77	76.13 ± 7.66,76–82	0.929	< 0.001	0.91–0.948	0.932	0.017	0.879–0.988
Gender (male%)		537 (83.91%)	172 (77.83%)	1.485	0.042	1.015–2.174	1.044	0.937	0.36–3.027
Bilateral Hematoma		134 (20.94%)	63 (28.51%)	0.664	0.021	0.469–0.941	0.698	0.474	0.261–1.869
Average mRS at admission (*n* ± SD), interquartile range		2.52 ± 1.06,2–3	3.99 ± 0.54,4–4	0.109	< 0.001	0.076–0.156	0.104	< 0.001	0.045–0.24
Average GCS at admission (*n* ± SD), interquartile range		13.45 ± 1.23,12–14	9.91 ± 1.12,9–11	15.071	< 0.001	9.449–24.038	11.752	< 0.001	7.033–19.637
Main clinical	Headache	341 (53.28%)	73 (33.03%)	2.312	< 0.001	1.678–3.186	2.098	0.137	0.791–5.564
symptoms at	Dyskinesia	279 (43.59%)	127 (57.47%)	0.572	< 0.001	0.42–0.779	1.268	0.626	0.489–3.288
admission	Dizziness	219 (34.22%)	55 (24.89%)	1.57	0.011	1.111–2.219	0.651	0.386	0.247–1.718
Treatment^#^	Burr-hole craniotomy	580 (90.63%)	180 (81.45%)		0.001	–		0.051	–
	Craniotomy	27 (4.22%)	16 (7.24%)	0.524	0.048	0.276–0.994	1.144	0.875	0.214–6.117
	Conservative treatment	33 (5.16%)	25 (11.31%)	0.41	0.001	0.237–0.707	4.091	0.015	1.318–12.694
Etiology	History of head trauma	349 (54.53%)	104 (47.06%)	1.349	0.055	0.993–1.833	0.896	0.802	0.381–2.109
Main	Cardio-cerebrovascular system disease	330 (51.56%)	146 (66.06%)	0.547	< 0.001	0.398–0.752	1.711	0.324	0.588–4.977
concomitant	Respiratory system disease	224 (35.00%)	122 (55.2%)	0.437	< 0.001	0.32–0.596	0.357	0.049	0.128–0.994
diseases at	Endocrine system disease	61 (9.53%)	28 (12.67%)	0.726	0.188	0.451–1.169	2.112	0.249	0.593–7.52
admission									

**TABLE 4c:** Factors influencing functional outcomes in young/middle group (*n* = 420).

**Variable (*n* = 420)**		**Favorable mRS (0–2), (*n* = 366)**	**Unfavorable mRS (3–6), (*n* = 54)**	**OR^∗^**	***p*-value*^∗^***	**95% CI^∗^**	**OR^∗∗^**	***p*-value^∗∗^**	**95% CI^∗∗^**
Mean age years ± SD, interquartile range (years)		47.96 ± 9.36,43–55	47.80 ± 10.55,41.5–56	1.002	0.904	0.972–1.032	1.001	0.961	0.952–1.053
Gender (male%)		333 (79.29%)	47 (87.04%)	1.503	0.359	0.629–3.591	3.276	0.157	0.632–16.974
Bilateral hematoma		75 (17.86%)	8 (14.81%)	1.482	0.331	0.671–3.274	1.314	0.703	0.323–5.351
Average mRS at admission (*n* ± SD), interquartile range		2.30 ± 1.01,1–3	3.93 ± 0.57,4–4	0.087	0	0.044–0.172	0.097	< 0.001	0.032–0.294
Average GCS at admission (*n* ± SD), interquartile range		13.24 ± 1.38,12–15	10.07 ± 0.92,9–11	5.245	0	3.552–7.745	3.919	< 0.001	2.431–6.317
Main clinical	Headache	296 (70.48%)	40 (74.07%)	1.48	0.246	0.763–2.869	0.431	0.172	0.129–1.443
symptoms at	Dyskinesia	57 (13.57%)	10 (18.52%)	0.812	0.582	0.386–1.705	0.299	0.142	0.06–1.497
admission	Dizziness	144 (34.29%)	17 (31.48%)	1.412	0.269	0.766–2.602	0.758	0.65	0.228–2.518
Treatment^#^	Burr-hole craniotomy	329 (89.89%)	43 (79.62%)		0.092	–		0.588	–
	Craniotomy	15 (4.10%)	4 (7.41%)	0.49	0.223	0.156–1.545	0.422	0.451	0.045–3.979
	Conservative treatment	22 (6.01%)	7 (12.96%)	0.411	0.055	0.166–1.018	1.565	0.591	0.306–8.001
Etiology	History of head trauma	256 (60.95%)	29 (53.7%)	2.006	0.019	1.124–3.582	1.219	0.712	0.426–3.49
Main	Cardio-cerebrovascular system disease	39 (9.29%)	12 (22.22%)	0.417	0.018	0.203–0.86	0.908	0.895	0.214–3.849
concomitant	Respiratory system disease	2 (0.48%)	3 (5.56%)	0.093	0.01	0.015–0.572	0.007	0.098	0–2.501
diseases at	Endocrine system disease	12 (2.86%)	4 (7.41%)	0.424	0.15	0.132–1.365	0.905	0.932	0.09–9.064
admission									

*All results are given as number (%) or mean ± SD. All data were calculated with logistic regression. The ORs are for favorable functional outcome. ^#^Burr-hole craniotomy is categorical variable, Craniotomy and Conservative treatment were dummy variables. ^∗^These results were from univariable analysis. ^∗∗^These results were from multivariable analysis.*

**TABLE 5:** Factors influencing functional outcomes in patients treated by conservative treatment (*n* = 87).

**Variable (*n* = 87)**		**Favorable mRS (0–2), (*n* = 55)**	**Unfavorable mRS (3–6), (*n* = 32)**	**OR^∗^**	***p*-value*^∗^***	**95% CI^∗^**	**OR^∗∗^**	***p*-value^∗∗^**	**95% CI^∗∗^**
Mean age years ± SD, interquartile range (years)		62.15 ± 17.53,51–78	70.68 ± 13.92,65–82	0.966	0.027	0.938–0.996	0.966	0.297	0.905–1.031
Gender (male%)		43 (78.18%)	22 (68.75%)	1.629	0.331	0.609–4.357	1.071	0.939	0.187–6.137
Bilateral Hematoma		12 (21.82%)	8 (25.00%)	0.837	0.734	0.301–2.332	0.582	0.555	0.096–3.51
Average mRS at admission (*n* ± SD), interquartile range		3.2 ± 0.75,3–4	4.13 ± 0.74,4–5	0.164	<0.001	0.069–0.389	0.053	0.001	0.009–0.308
Average GCS at admission (*n* ± SD), interquartile range		12.27 ± 1.81,11–14	10.22 ± 1.69,9–11	1.936	<0.001	1.401–2.675	2.645	0.002	1.441–4.855
Main clinical	Headache	34 (61.82%)	14 (43.75%)	2.082	0.105	0.859–5.045	3.527	0.186	0.545–22.846
symptoms at	Dyskinesia	13 (23.64%)	6 (18.75%)	1.341	0.595	0.454–3.965	2.208	0.559	0.154–31.578
admission	Dizziness	18 (32.73%)	15 (46.88%)	0.551	0.192	0.226–1.348	0.08	0.014	0.011–0.603
Treatment	Use of atorvastatin	28 (50.91%)	17 (53.13%)	0.915	0.842	0.382–2.19	7.464	0.045	1.041–53.507
Etiology	History of head trauma	31 (56.36%)	13 (40.63%)	1.888	0.159	0.78–4.57	1.581	0.56	0.339–7.363
Main	Cardio-cerebrovascular system disease	21 (38.18%)	20 (62.5%)	0.371	0.03	0.151–0.911	2.647	0.321	0.387–18.121
concomitant	Respiratory system disease	15 (27.27%)	15 (46.88%)	0.425	0.066	0.171–1.059	0.248	0.15	0.037–1.653
diseases at	Endocrine system disease	4 (7.27%)	4 (12.5%)	0.549	0.421	0.127–2.365	0.175	0.313	0.006–5.176
admission									

*All results are given as number (%) or mean ± SD. All data were calculated using logistic regression. The ORs showed favorable functional outcome. ^∗^These results were from univariable analysis. ^∗∗^These results were from multivariable analysis.*

Age and respiratory system diseases showed correlation with unfavorable outcome (*p* = 0.003, OR 0.963; *p* = 0.035, OR 0.419, respectively) in all patients, and similarly were significant in old group (*p* = 0.017, OR 0.932; *p* = 0.049, OR 0.357, respectively). In addition, the mRS at admission in conservative treatment patients was higher than the burr-hole surgeries patients analyzed by Chi Square test (*p* < 0.001), which indicates the patients of conservative treatments might have more serious clinical symptoms. Moreover, the prognosis of old subgroup receiving conservative treatment was better than those receiving the burr-hole drainage treatment (*p* < 0.015, OR 4.091). Generally, the higher GCS and lower mRS scores at admission predicted a favorable neurological outcome at discharge (*p* < 0.001, OR 7.481 and *p* < 0.001, OR 0.138, respectively) (Tables 4a,b).

The conservative treatments were performed in 87 patients, and these patients were further sub-classified into atorvastatin (20 mg daily for 1–6 months) plus symptomatic treatment group and symptomatic treatment group. Logistic regression in these patients was performed to predict the different outcomes between the two different conservative treatment subgroups. A sharp promotion of outcome was observed with the use of atorvastatin (*p* = 0.045, OR 7.464). In both groups, mRS was higher, GCS was lower and dizziness predicted the poor outcome (*p* = 0.001, OR 0.053 and *p* = 0.002, OR 2.645 and *p* = 0.008, OR 0.014, respectively) (Table 5).

## Discussion

We retrospectively collected the data of 1281 CSDH patients from two tertiary hospitals in China, and several novel observations were made after data analysis.

This is the first large retrospective report on the demographic description and short-term outcome features analysis of CSDH patients based on Chinese population. Recently, two large sample size researches were published by Japan and England. One was a retrospective report of more than 60 thousand patients of CSDH that resulted from head trauma in Japan, and described age-based etiology, treatment outcomes and post-operational recurrent rate (Toi et al., 2018). Another was a prospective study published by English, which reported the general features of 1205 CSDH patients, and mainly focused on the association between surgical treatment measurement and outcome (Brennan et al., 2017). Our results were obtained from Chinese patients, which might be a little different from the other races. It could help to fill up the data of CSDH patients in the whole world.

Our analysis results mainly showed that the dominant CSDH patients were males, with a median age of 60 years. Interestingly, in patients who were older than 40 years, the male-female ratio showed a decreased tendency from 91.47 to 68.42% with increasing age. We first described the symptoms that frequently occurred, which included headache, dyskinesia and dizziness. The neurological impairment assayed by GCS and mRS indicated that chronic brain injury in most of the CSDH patients was mild to moderate. The description of neuroimages revealed that almost 80% patients had unilateral hematomas, which was consistent with the published study (Huang et al., 2013). Different from the concept that the congenital intracranial structural abnormality was the most important etiological factor of CSDH, our research revealed that head trauma was still the main cause of CSDH, and less than 50% patients had no determined etiological cause. In addition, the young/middle aged patient seems to more likely meet head trauma, which was similar to the Mass’s report (Maas et al., 2017). The rest of the patients either might forget whether a head trauma occurred or not sure what happened before CSDH diagnosis. Combing these facts and considering the fact that more than 60 thousand CSDH patients in Japanese research resulted from brain trauma (Toi et al., 2018), it seemed that the congenital intracranial structural abnormality increases the risk of CSDH, but not the etiological cause. The concomitant diseases of CSDH were also considered the most often gerontological diseases like the cardio-cerebrovascular and respiratory system diseases. This seemed that although some CSDH patients still kept a clear mind, but their motor function was limited and the mRS might be more suitable for evaluate CSDH patients’ situation.

Secondly, we reported the differences of demographic and outcome features in different aged population. In the sub-group analysis, headache was more often in younger patients. This might be due to that the intracranial space in younger patients is limited because of their full brain volume. A small intracranial hematoma might be a silent mass in aged patients, but could cause severe hyper intracranial pressure (HIP) in a young man. Headache has thus become the most common neurological symptom in younger patients with CSDH as it ranks as the most common symptom in patients with HIP (Friedman, 2018). Meanwhile, dyskinesia mainly occurred in elderly patients, which always resulted from the injury to the motion area. The motion area is a prominent area in the cerebral surface. The injury to the motor area might be the result of persisting cortex suppression from the silent hematoma in aged CSDH patients.

In the outcome analysis, our study demonstrated that the respiratory system disease but not cardio-cerebrovascular disease was the strongest negative factor for unfavorable outcome in aged patients. The reason for this might be that the CSDH patients are almost aged and respiratory system disease is one of the most commonly observed diseases in the aged population in China. The respiratory system diseases included pneumonia, bronchitis, COPD and asthma, and COPD was the major of these. Hypoxemia is mostly prevalent in COPD (Rossi et al., 2002; Wang et al., 2011). Though hypoxemia could induce the enlargement of CSDH by aggravating angiogenesis disorder and inflammation (Krzystek-Korpacka et al., 2008; Dewan et al., 2015), our patients with very poor outcome were mostly resulted from a direct lung infection after receiving either burr-hole drainage or other therapies, and rarely occurs from an expansile CSDH. This indicated that anti-infective treatment selection might play a key role for the outcome.

Thirdly, we considered conservative treatment to be another choice that benefits some patients. Although primary treatment for CSDH in our study was the burr-hole drainage, which was recognized as the first-line treatment for CSDH, our study also revealed that the conservative treatment might be a better choice for some patients. The outcome analysis did not reveal the advantage of burr-hole drainage or conservative treatment when they are compared. However, conservative treatment based on atorvastatin seemed to benefit patients. The symptomatic treatment is the main conservative treatment for CSDH including headache relief treatment with osmotic therapy before 2014. Our team attempted atorvastatin treatment with a dose of 20 mg daily for 1–6 months to CSDH patients in 2014 (Wang et al., 2014), confirming the efficacy and safety by multi-center RCT in 2018 (Jiang et al., 2018). Since 2014, atorvastatin plus symptomatic treatment have been used in some of the neurosurgical departments in China. Hence, our study enrolled some patients who were treated with atorvastatin from 2014 to 2016.

However, there are some limitations in our study. Firstly, the presenting data were obtained from two medical centers only in China. But both medical centers recruited most of the patients in their geographic area, representing the top neurosurgical center in southwest and north China. So, it may partially represent the whole CSDH population in these areas of China. Next, we will acquire more data across the country. Secondly, there lacked the original neuroimaging and the surgical treatment protocol records in this study, hindering further analysis of this disease. Thirdly, this is a retrospective study without long-term follow-up outcome data and recurrence rates. Hence, further prospective research should be established with a better follow-up system in order to get more precise results. Fourthly, the patient number in this study who underwent treatment with atorvastatin was too small to draw the strongly convincing conclusion that atorvastatin benefited the patient.

## Conclusion

Chronic subdural hematoma mostly occurs in aged and male population with head trauma history of more than 50% patients. The top clinical symptoms are headache and dyskinesia, while headache is more often encountered in younger and dyskinesia in elderly patients. The respiratory disease might be the most significant concomitant disease that plays a negative role in CSDH treatment. It seems that conservative treatment might benefited some patients. Further research is required to confirm this conclusion.

## Data Availability Statement

The raw data supporting the conclusion of this manuscript will be made available by the authors, without undue reservation, to any qualified researcher.

## Author Contributions

RJ, JZ, and HF designed this study. JH, YT, and YS carried out this study, collected and analyzed the data, and contributed equally to this study. JH, YS, RH, SZ, HF, ZG, XL, and HL helped in data collection. YT, DW, and CG helped to analyze the results. JH wrote the draft. JZ and RJ revised the final version of the manuscript.

## Conflict of Interest

The authors declare that the research was conducted in the absence of any commercial or financial relationships that could be construed as a potential conflict of interest.
